# Improvement of Gait after Robotic-Assisted Training in Children with Cerebral Palsy: Are We Heading in the Right Direction?

**DOI:** 10.3390/medsci10040059

**Published:** 2022-10-13

**Authors:** Rosaria De Luca, Mirjam Bonanno, Carmela Settimo, Rosalia Muratore, Rocco Salvatore Calabrò

**Affiliations:** IRCCS Centro Neurolesi “Bonino Pulejo”, 98124 Messina, Italy

**Keywords:** ataxic-spastic, cerebral palsy, Lokomat, gross motor function

## Abstract

Cerebral palsy (CP) is a non-progressive congenital neurological disorder that affects different physical and cognitive functions in children. In addition to standard rehabilitation, advanced robotic gait devices are novel tools that are becoming progressively more common as part of the treatment of CP. The aim of this study is to evaluate the effects of Lokomat training, in addition to conventional rehabilitation, on the motor function and quality of life of children with ataxic-spastic CP (ASCP). Ten children with ASCP who attended the Robotic Rehabilitation OutClinic of the IRCCS Centro Neurolesi “Bonino Pulejo”, from April to June 2019, were enrolled in this study. They received twenty-four robotic rehabilitation sessions, twice a week for three months, each session lasting about 45 min. They were also provided with conventional physical and occupational therapy. After the innovative training, we found significant changes in the children’s outcomes, i.e., in GMFM (*p* < 0.001), with significant improvements in sitting (*p* < 0.03) and walking (*p* < 0.03). Moreover, the quality of life of the young patients, evaluated by their parents, significantly improved (*p* < 0.005). The use of robotic systems could be considered to be an effective complementary treatment to improve gait, as well as quality of life, in children with CP.

## 1. Introduction

Cerebral palsy (CP) is a congenital neurological disorder that affects different physical and cognitive functions in children. In detail, CP is “a group of permanent disorders that affect the development of movement and posture and cause activity limitation, which are attributed to non-progressive disturbances that have occurred in the developing fetal or infant brain” [1]. The underlying etiology of CP ranges from brain malformations to preterm white matter injury; hypoxic-ischemic injury; pre-, peri-, or postnatal stroke; genetic disorders; CNS infection; or early traumatic brain injury [2]. CP is the most common cause of physical disability in children, with a prevalence of 2–3/1000 live births worldwide [3]. Historically, CP has been diagnosed between the ages of 12 and 24 but, currently, diagnoses can be formulated faster at 6 months [4]. Brain damage related to CP is responsible for serious outcomes, such as walking limitations, muscle weakness, and reduced postural control and coordination, contributing to major difficulties in daily life activities. The clinical presentation of motor symptoms in CP varies a lot, and there are several classifications of those symptoms. About 90% of children with CP have gait alterations, such as ataxic walk [5,6], with a decrease in walking speed and reduced endurance [7], as well as decreased cardiorespiratory performance [8]. Therefore, autonomous walking and gait quality are often the goal of therapeutic treatments for CP children, to allow their autonomy in activities of daily life [9,10]. However, today there is no standardized protocol for treating CP physical symptoms [11]. The rehabilitative choice is related to different factors: the presence or not of spasticity, respiratory dysfunctions, and level and type of motor alterations. Neuromotor techniques, including Kabat and Bobath, as well as medical treatments such as botulinum toxin, are aimed at strengthening muscle, increasing joint range, reducing stiffness and spasticity, and improving coordination and balance [12]. Moreover, respiratory muscle training (RMT), a technique that aims to improve the function of the respiratory muscles through specific exercises, is fundamental to increase the endurance of patients [13]. In this context, robotic gait devices are novel tools that are starting to become progressively more common as part of the treatment for CP [14]. In particular, lower limb motor dysfunctions, with regard to gait abnormalities, negatively affect patient’s autonomy, and then their quality of life. Although different robotic devices with a fixed or overground design exist, there is no consensus about their use and effectiveness in CP [15,16]. Nevertheless, it is evident that they could be a promising complement treatment to standard neurorehabilitation, even though there is poor evidence to obtain decisive results on the efficacy of these robotic tools [17]. 

The purpose of this study is to evaluate the potential effects of Lokomat training, in addition to conventional rehabilitation, on the gross motor functions and quality of life of ataxic-spastic children with CP.

## 2. Materials and Methods

Children with ataxic-spastic cerebral palsy (ASCP) syndrome who attended the Robotic Rehabilitation Outpatient Clinic of IRCCS Centro Neurolesi “Bonino Pulejo”, from April to June 2019, were screened for inclusion in this study. Ten children (9 males and 1 female) out of 30, with a mean age of 8.6 (±2.31), were enrolled. In this study, we included children with a diagnosis of bilateral ASCP, aged six to twelve years, and with good therapeutic compliance; moreover, to be included they had to be first users of the robotic device. 

We excluded patients with severe mental retardation (QI < 40), psychiatric symptoms, sensory deficits and severe spasticity (Ashworth > 3), severe motor deficit, and other medical problems potentially affecting the experimental training. Children on new pharmacological treatments, in the last 6 months, such as botox and baclofen, were also excluded, as well as those classified as Gross Motor Function Classification System Level V (i.e., children limited in their ability to maintain antigravity head and trunk postures and control leg and arm movements).

All caregivers gave their written informed consent for study participation and data publication. The study was approved by the local ethics committee (IRCCSME 42/18).

**Figure 1:**
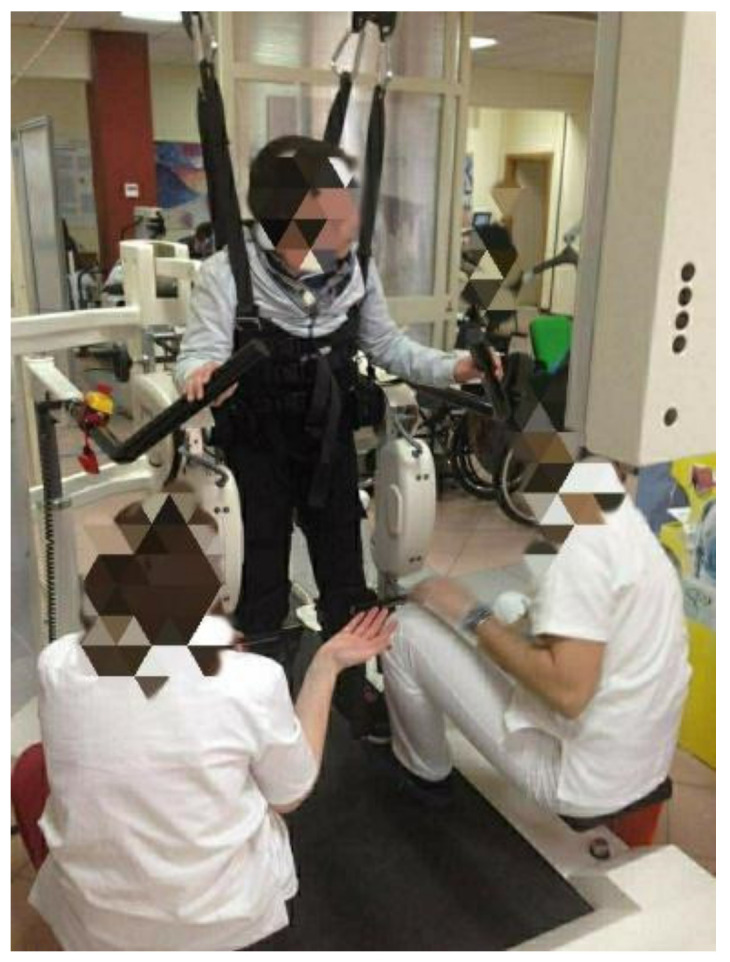
CP child is supported by the use of the Lokomat robotic device during a gait training session.

Children with ASCP received 24 robotic rehabilitation sessions, 2 times per week for a total of 3 months, and each session lasted up to 45 min. The robotic rehabilitative session was performed using the Lokomat device with the pediatric module. Throughout the training program, the Lokomat sessions were individually set according to the functional level of each patient. When the session was perceived to be too difficult, a slight change in settings (e.g., decrease speed or increase robot’s guidance) was made to allow the participant to perform the training. The Lokomat is an emergent robotic system that includes an advanced gait orthosis with computer-controlled linear actuators at each hip and knee joint, a body weight support, and a treadmill (Figure 1). Gait pattern and guidance force were individually adapted to children’s needs, finalized to improve the functional abilities in the sagittal, frontal, and transverse planes. Guidance force was initially set at 100% and body weight support at 80%, and then individually increased according to the improvement. The speed was set to the maximum walking speed tolerated by the patient, starting at 0.8 speed in order to avoid an increase in spasticity potentially interfering with the training session. Each Lokomat training session was combined with task-oriented exercises (e.g., step over an obstacle, kick a ball) and biofeedback to increase the patients’ motivation and promote their active participation.

Each pediatric Lokomat training (PLT) session was carried out by a multi-specialist rehabilitative team, including a neurologist, psychiatric therapist, physiotherapist, and neuropsychomotricist. In addition to robotics, all children were submitted to conventional physical and occupational therapy, consisting of ground activities according to Kabat techniques that promoted children’s straightening and balance and Bohath techniques that focused on head control, rolling, and preparation for sitting. Finally, to ensure adequate therapeutic compliance, the therapists created playful settings to ease ASCP children performing conventional physiotherapy exercises. 

At T0 and T1 (pre- and post-PLT), each participant’s motor function was evaluated using the GMFM, the clinical assessors (who were different neuropsychomotricists from those who performed the training), were familiar with this tool. The GMFM-88 consists of 88 items, divided into five categories (lying and rolling; sitting; crawling and kneeling; standing; walking, running, and jumping). Each item is scored on a four-point Likert scale. The tool has been validated in children with CP from 5 months to 16 years of age [18,19]. The total score of the GMFM-88 is calculated using a score for all dimensions or specific dimension(s) of interest. A 5-year old child without motor disabilities is able to reach the maximum score. Moreover, the effect of the functional recovery on quality of life was investigated using the Cerebral Palsy Quality of Life Questionnaire (CP QOL), a parent-proxy measure for children aged from 4 to 12 years [20]. 

We performed a descriptive statistical analysis of means, medians, and standard deviation (see Table 1), administering the Shapiro–Wilk test to verify the normality of the small sample. The sampling distribution was not normal and we conducted a Wilcoxon signed rank test to compare scores in the pre- and post-PLT, using the software R 4.1.3 [21]. A *p* < 0.05 was considered to be significance level. In addition, we calculated the effect size (ES) using Glass’s delta, preferable for non-parametric and small sample sizes.

## 3. Results

By comparing all of the means of the clinical test scores between baseline (T0) and follow-up (T1), we found significant changes in ASCP children’s outcomes of the GMFM (*p* < 0.001); in particular, by analyzing the five dimensions, we observed a significant improvement in sitting (*p* < 0.03) as well as walking, running, and jumping (E dimension) (*p* < 0.03), specifically a large ES was found for walking (*p* < 0.03, ES = 0.82), as shown in Table 1. 

In addition, the quality of life of patients with CP, based on the GMFM administered to the caregivers, showed a significant improvement at the end of the PLT (*p* < 0.005, ES = 1.14). Moreover, we performed a statistical analysis without the data from the only female child on the GMFM, in order to avoid influences on results. However, there were no significant changes to report, except for better ES values in the rolling (ES = 0.40) and sitting (ES = 0.43) GMFM’s dimensions.

## 4. Discussion

Robotic systems are new devices that are becoming increasingly popular as part of the treatment for CP. In line with the current literature [22,23], our data showed that combined training, using conventional and advanced methods (by means of the Lokomat) can be useful to optimize the gross motor functions of ASCP children, especially in walking [24], and to a lesser extent in standing and sitting. Indeed, large ES values reflecting clinical improvement were found in the walking dimension as the main motor function potentiated by the Lokomat training. However, after excluding female data, we found close to medium ES in sitting and rolling as a result of improved head and trunk control. 

Moreover, the improvement in gait function positively affected children’s QOL, confirmed by a large effect size (see Table 1). According to our opinion, implementing PLT in the current outpatient activity could be useful to also improve ASCP ataxic symptoms, such as uncoordinated gait and mobility difficulties in postural changes. In fact, we suppose that the main advantage of also using neurorobotics in neurorehabilitation relies on the potentially strong effect in “modulating” cortical plasticity and cerebello-motor connectivity through augmented sensorial feedback and the use of virtual reality [25] integrated into the Lokomat device [26,27]. Robotic systems are emerging rehabilitative tools that are being used in addition to standard sensory-motor training for children affected by CP. The objective of robotic systems is to help patients to achieve correct motor function, as these tools can provide high intensity, repetitive, task-specific, and interactive training [28]. In addition to our findings, the current literature suggests that systematic use of Lokomat devices can also decrease spasticity and improve joint amplitudes, autonomy, muscle tone, strength, etc. [28,29]. However, as these are such new techniques, their feasibility and effectiveness in the treatment of CP are still controversial, and we have not investigated this issue. Van Hedel et al. included patients with neurological disorders and gait difficulties (cerebral palsy, stroke injury, spinal cord injury, traumatic brain injury, etc.) who were using a Lokomat system as part of their treatment in order to draw conclusions about its use and even extended its use to other diagnoses. However, so far, no relevant conclusions have been obtained [30]. Several controlled trials have shown superior effects of Lokomat in acute stroke patients with respect to walking ability and gait velocity (patients walk more symmetrically, and higher velocities result in a facilitation of paretic muscles and render gait more efficient) [31,32]. These findings should be replicated in the CP population.

There is some evidence that more severely impaired patients with stroke might improve more than less affected patients [33]. Similar results have been found for children with CP [34], although these data are controversial [35,36]. Other attempts to find correlations between responsiveness and diagnostic factors in stroke [37,38,39] and spinal cord injury [40] have not been very successful. We believe that the main purpose of robotic devices is to support CP patients to promote a better gait through the implementation of this modern robotic goal-oriented therapy in current clinical practice. Indeed, given the features of the Lokomat system, the training may lead to an improvement of postural and antigravity muscles (including glutes and quadriceps) and this could explain why the walking item showed better improvement.

Our study has some limitations to acknowledge. The small sample size prevented us from generalizing the results to the larger CP population. Nonetheless, all children were affected by bilateral ataxic CP, and therefore, the results confirmed the idea that the Lokomat may work on ataxic gait, as has been demonstrated in other neurological disorders [41].

The analysis was performed on available data derived from a retrospective study with a few patients; then, to perform correlations between the level of disability, spasticity, and the improvements in gait were not possible, as well as analyzing the difference between the children that presented better results and the children that presented worse results. Prospective randomized clinical trials are needed to investigate these important issues. It could also be useful to investigate the clinical long-term effects using different and more objective outcome measures, including gait analysis tools, to confirm our promising data.

A strength of the study is that the sample was homogeneous since it was composed of patients with bilateral CP and ataxia, which is a significant problem affecting functional recovery in such patient population. Indeed, as far as we know, this is the first time that the Lokomat system has been successfully used to improve the disabling symptoms in children with CP. However, further studies should be fostered to confirm our promising results.

## 5. Conclusions

In spite of the poor evidence that was shown in the literature and the controversies, our findings support the idea that PLT could be effective in improving gait abilities (as per the walking item of the GMFM), and then QoL in individuals with CP. However, the use of robotic devices cannot be applied as a treatment alone, but as an additional advanced and complementary training to CP standard neurorehabilitation. More studies with larger samples, higher quality methodology (including deeper statistical analysis), and long-term follow-up with other specific outcome measures are needed to confirm the beneficial effect of these new technologies in CP clinical practice.

## Figures and Tables

**Table 1:** Statistical analysis of Gross Motor Function Measure and Cerebral Palsy Quality of Life ASCP’s scores.

GMFM Dimension’s Questionnaire	Means		Standard Deviation		Median		*p*-Value *	ES
	T0	T1	T0	T1	T0	T1		
Total score	56.58	59.31	28.74	27.02	58.25	59	<0.001	0.09
A. Lying and rolling Lying	89.42	92.35	22.38	14.64	100	100	0.37	0.13
	60.71	60.87	6.72	6.24	60	61	0.37	0.02
Rolling	35.5	39.28	12.38	6.72	37.5	40	1	0.30
B. Sitting	75	78.17	32.19	28.84	87.5	88.35	<0.03	0.09
C. Crawling/kneeling	51.9	55.96	44.9	41.15	58.35	60.7	0.10	0.09
D. Standing	37.7	39.49	32.39	34.40	39.75	39.75	0.097	0.07
E. Walking, running and jumping	28.85	31.09	28.32	29.52	23.45	24.3	<0.03	0.07
Walking	11	26.66	19.05	46.18	0	0	<0.03	0.82
Running	0	0.33	1	0	0	0	NA	NA
Jumping	0	0	0	0	0	0	NA	NA
CP QOL	40	52.1	10.54	11.23	45	55	<0.005	1.14
	T0 score	T1 score	Percentage of improvement					
	35	40	5%					
	25	35	10%					
	45	55	5%					
	25	40	15%					
	55	60	5%					
	45	55	10%					
	30	45	15%					
	45	66	21%					
	45	60	15%					
	50	60	10%					

Legend: ES (effect size) calculates with Glass’s delta analysis. ES: 0.2 = small effect; 0.5 = medium effect; 0.8 = large effect. * *p* < 0.05.

## Data Availability

Data are available on-demand to the corresponding author.
